# Supplementary material for: Large-Scale Assessment of NF1 Single Amino Acid Variants as HLA Class I Neoantigens
Source: bioRxiv. 2026 May 13:2026.05.10.724138. Preprint. [Version 1] doi: 10.64898/2026.05.10.724138 (PMC13175214; doi:10.64898/2026.05.10.724138)
Supplement: Supplement 2 [file media-2.pdf]

NF1 Construct 1-8

Construct ID: NF1_C1

Amino acid sequence

**MRVTAPRTLILLLSGALALTETWAGSGGSGGGGSGGPGIDEETSEESLLTPTSPYLPALQSQLSITANGGSGGG
GSGGAIVSPYEAGILDKKPPPRIERSLKLMSKILQSIANHVLGGSGGGGSGGQRILYEYLAEASVVLPKVFPVV
HNLLDSKINTLLSLCQGGSGGGGSGGGKLAEHIEHEQQKLPAAILALEEDLKVFHNALKGGSGGGGSGGAGQPK
DTMRLDETMLAKQLLPEICHFLHTCREGGSLGGGGSGIVGIVAGLAVLAVVVIGAVVATVMCRRKSSGGKGGSY
SQAASSDSAQGSDVSLTA***

Nucleotide sequence

TGGCTAGTTAAGCTTGGATCCGCCACC**ATG**CGGGTGACTGCTCCACGGACACTGATTCTGCTTCTGTCAGGGGC
CTTGGCGCTCACCGAGACTTGGGCCGGATCAGGAGGCTCTGGAGGCGGAGGTTCTGGAGGCCCTGGAATTGATG
AAGAAACCAGTGAAGAATCCCTCCTGACTCCCACATCTCCTTACCTTCCTGCACTGCAGAGCCAGCTTAGTATC
ACTGCCAACGGAGGTAGCGGAGGCGGGGGTTCCGGCGGAGCCATTGTCTCACCGTATGAAGCAGGGATTTTAGA
TAAAAAGCCACCACCTAGAATCGAAAGGAGCTTGAAGTTAATGTCAAAGATACTTCAGAGTATTGCCAATCATG
TTCTCGGCGGGTCTGGAGGCGGGGGCTCAGGAGGCCAACGAATTCTTTATGAATACTTAGCAGAGGCCAGTGTT
GTGTTGCCCAAAGTCTTTCCTGTTGTGCATAATTTGTTGGACTCTAAGATCAACACCCTGTTATCATTGTGCCA
AGGTGGGAGTGGGGGTGGGGGAAGCGGCGGCGGGAAACTGGCTGAGCACATAGAGCATGAACAACAGAAACTAC
CTGCTGCCATCTTGGCTTTAGAAGAGGACCTGAAGGTATTCCACAATGCTCTCAAGGGGGGGTCCGGAGGCGGC
GGCTCCGGGGGCGCTGGGCAACCAAAGGACACAATGAGATTAGATGAAACGATGCTGGCCAAACAGTTGCTGCC
AGAAATCTGCCATTTTCTTCACACCTGTCGTGAAGGTGGATCTCTCGGAGGAGGAGGGAGCGGCATCGTCGGCA
TCGTCGCAGGCCTTGCGGTTCTGGCGGTCGTTGTCATCGGAGCAGTTGTGGCTACTGTCATGTGCAGGCGCAAA
AGCAGCGGCGGCAAAGGAGGTAGTTATTCACAGGCCGCCTCATCCGACAGTGCCCAAGGCTCCGATGTCTCACT
CACCGCG**TGA**ATTCACCATTGAGTTTAAA

Construct ID: NF1_C2

Amino acid sequence

**MRVTAPRTLILLLSGALALTETWAGSGGSGGGGSGGKVIGRMCKIIDKTYLSPTPTLEQHLMWDDIAILGGSGG
GGSGGLPGIDEETSEESLLTPTSPYPPALQRQLSITANLNLSNGGSGGGGSGGYLPGIDEETSEEALLTPTSPY
PPALQSQLSITANLNLSGGSGGGGSGGLPLQPEEGDGVELMEAESQLFLKYFTLFMNLLNDGGSGGGGSGGQRI
LYEYLAEASVVFPKVFPVVHDLLDSKINTLLSLCQGGSLGGGGSGIVGIVAGLAVLAVVVIGAVVATVMCRRKS
SGGKGGSYSQAASSDSAQGSDVSLTA***

Nucleotide sequence

TGGCTAGTTAAGCTTGGATCCGCCACC**ATG**CGGGTGACTGCTCCACGGACACTGATTCTGCTTCTGTCAGGGGC
CTTGGCGCTCACCGAGACTTGGGCCGGATCAGGAGGCTCTGGAGGCGGAGGTTCTGGAGGCAAGGTTATTGGAA
GGATGTGCAAAATAATTGACAAGACATACTTATCTCCAACTCCTACTTTAGAACAACATCTTATGTGGGATGAT
ATTGCTATTTTAGGAGGTAGCGGAGGCGGGGGTTCCGGCGGACTGCCTGGAATTGATGAAGAAACCAGTGAAGA
ATCCCTCCTGACTCCCACATCTCCTTACCCTCCTGCACTGCAGAGGCAGCTTAGTATCACTGCCAACCTTAACC
TTTCTAATGGCGGGTCTGGAGGCGGGGGCTCAGGAGGCTACCTGCCTGGAATTGATGAAGAAACCAGTGAAGAA
GCCCTCCTGACTCCCACATCTCCTTACCCTCCTGCACTGCAGAGCCAGCTTAGTATCACTGCCAACCTTAACCT
TTCTGGTGGGAGTGGGGGTGGGGGAAGCGGCGGCCTCCCTCTGCAGCCTGAAGAAGGAGATGGTGTGGAATTGA

TGGAAGCCGAATCACAGTTATTTCTTAAATACTTCACATTATTTATGAACCTTTTGAATGACGGGGGGTCCGGA
GGCGGCGGCTCCGGGGGCCAACGAATCCTTTATGAATACTTAGCAGAGGCCAGTGTTGTGTTTCCCAAAGTCTT
TCCTGTTGTGCATGATTTGTTGGACTCTAAGATCAACACCCTGTTATCATTGTGCCAAGGTGGATCTCTCGGAG
GAGGAGGGAGCGGCATCGTCGGCATCGTCGCAGGCCTTGCGGTTCTGGCGGTCGTTGTCATCGGAGCAGTTGTG
GCTACTGTCATGTGCAGGCGCAAAAGCAGCGGCGGCAAAGGAGGTAGTTATTCACAGGCCGCCTCATCCGACAG
TGCCCAAGGCTCCGATGTCTCACTCACCGCG**TGA**ATTCACCATTGAGTTTAAA

Construct ID: NF1_C3

Amino acid sequence

**MRVTAPRTLILLLSGALALTETWAGSGGSGGGGSGGYLSQLIILDTLENCLAGQPKDTMRLDETMLVKQLLPEI
GGSGGGGSGGCRQAQTKLEVALYMFPWNPDTEAVLVAMSCFRHLCEEAGGSGGGGSGGSATGGLGSIKAEVMAH
TAVALASGNVKLVSSKVIGGSGGGGSGGLRIFNDKSPEEVCMAIRNPLEWHCKQMDHFVGGGSGGGGSGGELSQ
PDSIPQHTNIRPKDVPGTLLNIALLNLGSSDPSLGGSLGGGGSGIVGIVAGLAVLAVVVIGAVVATVMCRRKSS
GGKGGSYSQAASSDSAQGSDVSLTA***

Nucleotide sequence

TGGCTAGTTAAGCTTGGATCCGCCACC**ATG**CGGGTGACTGCTCCACGGACACTGATTCTGCTTCTGTCAGGGGC
CTTGGCGCTCACCGAGACTTGGGCCGGATCAGGAGGCTCTGGAGGCGGAGGTTCTGGAGGCTATCTCTCTCAGT
TGATTATATTGGATACACTGGAAAATTGTCTTGCTGGGCAACCAAAGGACACAATGAGATTAGATGAAACGATG
CTGGTCAAACAGTTGCTGCCAGAAATCGGAGGTAGCGGAGGCGGGGGTTCCGGCGGATGCCGACAAGCCCAGAC
CAAACTAGAAGTGGCCCTGTACATGTTTCCGTGGAACCCTGACACTGAAGCTGTTCTGGTTGCCATGTCCTGTT
TCCGCCACCTCTGTGAGGAAGCAGGCGGGTCTGGAGGCGGGGGCTCAGGAGGCAGTGCAACAGGTGGCTTGGGA
TCAATAAAAGCTGAGGTGATGGCACATACTGCTGTAGCTTTGGCTTCTGGAAATGTGAAATTGGTTTCAAGCAA
GGTTATTGGTGGGAGTGGGGGTGGGGGAAGCGGCGGCCTCCGTATATTCAATGACAAGAGTCCAGAGGAAGTAT
GTATGGCAATCCGGAATCCTCTGGAGTGGCACTGCAAGCAAATGGATCATTTTGTTGGAGGGGGGTCCGGAGGC
GGCGGCTCCGGGGGCGAACTGTCACAGCCCGACTCTATCCCCCAACACACCAATATTCGGCCAAAAGATGTCCC
TGGGACACTGCTCAATATCGCATTACTTAATTTAGGCAGTTCTGACCCGAGTTTAGGTGGATCTCTCGGAGGAG
GAGGGAGCGGCATCGTCGGCATCGTCGCAGGCCTTGCGGTTCTGGCGGTCGTTGTCATCGGAGCAGTTGTGGCT
ACTGTCATGTGCAGGCGCAAAAGCAGCGGCGGCAAAGGAGGTAGTTATTCACAGGCCGCCTCATCCGACAGTGC
CCAAGGCTCCGATGTCTCACTCACCGCG**TGA**ATTCACCATTGAGTTTAAA

Construct ID: NF1_C4

Amino acid sequence

**MRVTAPRTLILLLSGALALTETWAGSGGSGGGGSGGLSQPDSIPQHTKIRPKNVPGTLLNIALLNLGSSDPSLR
GGSGGGGSGGPSLRSAAYNLLCVLTCTFNLKIEGQLLETSGLGGSGGGGSGGETRSYKYLLLSMVKPIHADPKL
LLCNPRKQGPETQGSTGGSGGGGSGGLPLQPEEGDGVELMEAISQLFLKYFTLFMNLLNDGGSGGGGSGGNTEA
WEDTHAKWEQATKPILNYPKAKMEDGQAGGSLGGGGSGIVGIVAGLAVLAVVVIGAVVATVMCRRKSSGGKGGS
YSQAASSDSAQGSDVSLTA***

Nucleotide sequence

TGGCTAGTTAAGCTTGGATCCGCCACC**ATG**CGGGTGACTGCTCCACGGACACTGATTCTGCTTCTGTCAGGGGC
CTTGGCGCTCACCGAGACTTGGGCCGGATCAGGAGGCTCTGGAGGCGGAGGTTCTGGAGGCCTGTCACAGCCCG
ACTCTATCCCCCAACACACCAAGATTCGGCCAAAAAATGTCCCTGGGACACTGCTCAATATCGCATTACTTAAT
TTAGGCAGTTCTGACCCGAGTTTACGGGGAGGTAGCGGAGGCGGGGGTTCCGGCGGACCGAGTTTACGGTCAGC
TGCCTATAATCTTCTGTGTGTCTTAACTTGTACCTTTAATTTAAAAATCGAGGGCCAGTTACTAGAGACATCAG
GTTTAGGCGGGTCTGGAGGCGGGGGCTCAGGAGGCGAGACAAGAAGCTATAAGTATCTTCTCTTGTCCATGGTG
AAACCAATTCATGCAGATCCAAAGCTCTTGCTTTGTAATCCAAGAAAACAGGGGCCCGAAACCCAAGGCAGTAC
AGGTGGGAGTGGGGGTGGGGGAAGCGGCGGCCTCCCTCTGCAGCCTGAAGAAGGAGATGGTGTGGAATTGATGG
AAGCCATATCACAGTTATTTCTTAAATACTTCACATTATTTATGAACCTTTTGAATGACGGGGGGTCCGGAGGC
GGCGGCTCCGGGGGCAACACTGAGGCTTGGGAAGATACACATGCAAAATGGGAACAAGCAACAAAGCCAATCCT
TAACTATCCAAAAGCCAAAATGGAAGATGGCCAGGCTGGTGGATCTCTCGGAGGAGGAGGGAGCGGCATCGTCG
GCATCGTCGCAGGCCTTGCGGTTCTGGCGGTCGTTGTCATCGGAGCAGTTGTGGCTACTGTCATGTGCAGGCGC
AAAAGCAGCGGCGGCAAAGGAGGTAGTTATTCACAGGCCGCCTCATCCGACAGTGCCCAAGGCTCCGATGTCTC
ACTCACCGCG**TGA**ATTCACCATTGAGTTTAAA

Construct ID: NF1_C5

Amino acid sequence

MRVTAPRTLILLLSGALALTETWAGSGGSGGGGSGGGFSKSSIELKHLCLQYMTPWLSNLVRFCKHNDDGGSGG
GGSGGMLSFNNSLDVAAHLPYLFHVVTFLLATGPLSLRASTHGGGSGGGGSGGLPGIDEETSEESFLTPTSPYP
PALQSQLSITANLNLSNGGSGGGGSGGAGLPLQPEEGDGVELMEAKSQLFRKYFTLFMNLLNDCSGGSGGGGSG
GRISPHNNQHFKIYLAQNSPSTFHYVLVNSLHRIITNSAGGSLGGGGSGIVGIVAGLAVLAVVVIGAVVATVMC
RRKSSGGKGGSYSQAASSDSAQGSDVSLTA*

Nucleotide sequence

TGGCTAGTTAAGCTTGGATCCGCCACC**ATG**CGGGTGACTGCTCCACGGACACTGATTCTGCTTCTGTCAGGGGC
CTTGGCGCTCACCGAGACTTGGGCCGGATCAGGAGGCTCTGGAGGCGGAGGTTCTGGAGGCGGATTTAGCAAAT
CTAGTATTGAATTGAAACACCTTTGTTTGCAATACATGACTCCATGGCTGTCAAATCTAGTTCGTTTTTGCAAG
CATAATGATGATGGAGGTAGCGGAGGCGGGGGTTCCGGCGGAATGCTGTCCTTCAACAATTCCCTTGATGTGGC
AGCTCATCTTCCCTACCTCTTCCACGTTGTTACTTTCTTATTAGCCACAGGTCCGCTCTCCCTTAGAGCTTCCA
CACATGGAGGCGGGTCTGGAGGCGGGGGCTCAGGAGGCCTGCCTGGAATTGATGAAGAAACCAGTGAAGAATCC
TTCCTGACTCCCACATCTCCTTACCCTCCTGCACTGCAGAGCCAGCTTAGTATCACTGCCAACCTTAACCTTTC
TAATGGTGGGAGTGGGGGTGGGGGAAGCGGCGGCGCTGGTCTCCCTCTGCAGCCTGAAGAAGGAGATGGTGTGG
AATTGATGGAAGCCAAATCACAGTTATTTCGTAAATACTTCACATTATTTATGAACCTTTTGAATGACTGCAGT
GGGGGGTCCGGAGGCGGCGGCTCCGGGGGCCGTATAAGCCCTCACAACAACCAACACTTTAAGATCTACCTGGC
TCAGAAcTCACCTTCTACATTTCACTATGTGCTGGTAAATTCACTCCATCGAATCATCACCAATTCCGCAGGTG
GATCTCTCGGAGGAGGAGGGAGCGGCATCGTCGGCATCGTCGCAGGCCTTGCGGTTCTGGCGGTCGTTGTCATC
GGAGCAGTTGTGGCTACTGTCATGTGCAGGCGCAAAAGCAGCGGCGGCAAAGGAGGTAGTTATTCACAGGCCGC
CTCATCCGACAGTGCCCAAGGCTCCGATGTCTCACTCACCGCG**TGA**ATTCACCATTGAGTTTAAA

Construct ID: NF1_C6

Amino acid sequence

MRVTAPRTLILLLSGALALTETWAGSGGSGGGGSGGRYMLMLSFNNSLDVAAHPPYLFHVVTFLVATGPGGSGG
GGSGGILICRNKFLLKNKQADRSSCHFLLFCGVGCDIPSSGNTGGSGGGGSGGLSSTEILKWLREILICRNNFL
LKNKQADRSSCHFGGSGGGGSGGSVSESNVLLDEEVLTDPKIQVLLLTVLATLVKYTTDEFGGSGGGGSGGDVP
GTLLNIALLNLDSSDPSLRSAAYNLLCALTGGSLGGGGSGIVGIVAGLAVLAVVVIGAVVATVMCRRKSSGGKG
GSYSQAASSDSAQGSDVSLTA*

Nucleotide sequence

TGGCTAGTTAAGCTTGGATCCGCCACC**ATG**CGGGTGACTGCTCCACGGACACTGATTCTGCTTCTGTCAGGGGC
CTTGGCGCTCACCGAGACTTGGGCCGGATCAGGAGGCTCTGGAGGCGGAGGTTCTGGAGGCCGCTACATGCTGA
TGCTGTCCTTCAACAATTCCCTTGATGTGGCAGCTCATCCTCCCTACCTCTTCCACGTTGTTACTTTCTTAGTA
GCCACAGGTCCGGGAGGTAGCGGAGGCGGGGGTTCCGGCGGAATATTGATCTGCAGGAATAAATTTCTTCTTAA
AAATAAGCAGGCAGATAGAAGTTCCTGTCACTTTCTCCTTTTTTGCGGGGTAGGATGTGATATTCCTTCTAGTG
GAAATACCGGCGGGTCTGGAGGCGGGGGCTCAGGAGGCCTTAGTAGCACAGAAATTCTCAAGTGGTTGCGGGAA
ATATTGATCTGCAGGAATAACTTTCTTCTTAAAAATAAGCAGGCAGATAGAAGTTCCTGTCACTTTGGTGGGAG
TGGGGGTGGGGGAAGCGGCGGCTCAGTGTCTGAATCAAATGTTCTCTTGGATGAAGAAGTACTTACTGATCCGA
AGATCCAGGTGCTGCTTCTTACTGTTCTAGCTACACTGGTAAAATATACCACAGATGAGTTTGGGGGGTCCGGA
GGCGGCGGCTCCGGGGGCGATGTCCCTGGGACACTGCTCAATATCGCATTACTTAATTTAGACAGTTCTGACCC
GAGTTTACGGTCAGCTGCCTATAATCTTCTGTGTGCCTTAACTGGTGGATCTCTCGGAGGAGGAGGGAGCGGCA
TCGTCGGCATCGTCGCAGGCCTTGCGGTTCTGGCGGTCGTTGTCATCGGAGCAGTTGTGGCTACTGTCATGTGC
AGGCGCAAAAGCAGCGGCGGCAAAGGAGGTAGTTATTCACAGGCCGCCTCATCCGACAGTGCCCAAGGCTCCGA
TGTCTCACTCACCGCG**TGA**ATTCACCATTGAGTTTAAA

Construct ID: NF1_C7

Amino acid sequence

MRVTAPRTLILLLSGALALTETWAGSGGSGGGGSGGTSLETVTEALLEIMEACMREIPTCKWLDQWTELGGSGG
GGSGGLMHSIGLGYHKDLQTRATFMKVLTKILQQGTEFDTLAEGGSGGGGSGGPTSDAVNHSLSFISDGNVLVL
HRLLWNNQEKIGGGSGGGGSGGHAIQIKTKLCQLIEVMMARRDDLSFCQEMKFRNKMVEYGGSGGGGSGGLSST
EILKWLREILICRNKFLLKNNQADRSSCHFLLFGGSLGGGGSGIVGIVAGLAVLAVVVIGAVVATVMCRRKSSG
GKGGSYSQAASSDSAQGSDVSLTA*

Nucleotide sequence

TGGCTAGTTAAGCTTGGATCCGCCACC**ATG**CGGGTGACTGCTCCACGGACACTGATTCTGCTTCTGTCAGGGGC
CTTGGCGCTCACCGAGACTTGGGCCGGATCAGGAGGCTCTGGAGGCGGAGGTTCTGGAGGCACATCCTTGGAAA
CAGTCACAGAAGCTTTGTTGGAGATCATGGAGGCATGCATGAGAGAGATTCCAACGTGCAAGTGGCTGGACCAG
TGGACAGAACTAGGAGGTAGCGGAGGCGGGGGTTCCGGCGGACTCATGCACTCCATAGGCTTAGGTTACCACAA
GGATCTCCAGACAAGAGCTACATTTATGAAAGTTCTGACAAAAATCCTTCAACAAGGCACAGAATTTGACACAC
TTGCAGAAGGCGGGTCTGGAGGCGGGGGCTCAGGAGGCCCTACAAGTGATGCAGTAAATCATAGTCTTTCCTTC
ATAAGTGACGGCAATGTGCTTGTTTTACATCGTCTACTCTGGAACAATCAGGAGAAAATTGGGGGTGGGAGTGG
GGGTGGGGGAAGCGGCGGCCATGCAATTCAAATAAAAACGAAACTGTGTCAATTAATTGAAGTAATGATGGCAA
GGAGAGATGACCTCTCATTTTGCCAAGAGATGAAATTTAGGAATAAGATGGTAGAATACGGGGGGTCCGGAGGC
GGCGGCTCCGGGGGCCTTAGTAGCACAGAAATTCTCAAGTGGTTGCGGGAAATATTGATCTGCAGGAATAAATT
TCTTCTTAAAAATAACCAGGCAGATAGAAGTTCCTGTCACTTTCTCCTTTTTGGTGGATCTCTCGGAGGAGGAG
GGAGCGGCATCGTCGGCATCGTCGCAGGCCTTGCGGTTCTGGCGGTCGTTGTCATCGGAGCAGTTGTGGCTACT

GTCATGTGCAGGCGCAAAAGCAGCGGCGGCAAAGGAGGTAGTTATTCACAGGCCGCCTCATCCGACAGTGCCCA
AGGCTCCGATGTCTCACTCACCGCG**TGA**ATTCACCATTGAGTTTAAA

Construct ID: NF1_C8

Amino acid sequence

**MRVTAPRTLILLLSGALALTETWAGSGGSGGGGSGGLQYINVDCAKLKRLLKKTAFKFKALKKVAQLAVINSLG
GSGGGGSGGSLLAGLPLQPEEEDGVELMEAKSQLFLKYFTLFMNLLNGGSGGGGSGGHQECEAIVQSIIHMRTR
WELSQPDSIPQHTKIGGSGGGGSGGILICRNKFLLKNKQADRSSFHFLLFYGVGCDIPSSGGGSGGGGSGGEGY
LAATYPTVGQISPRARKSMSLDMGQPSQANTKKLGGSLGGGGSGIVGIVAGLAVLAVVVIGAVVATVMCRRKSS
GGKGGSYSQAASSDSAQGSDVSLTA***

Nucleotide sequence

TGGCTAGTTAAGCTTGGATCCGCCACC**ATG**CGGGTGACTGCTCCACGGACACTGATTCTGCTTCTGTCAGGGGC
CTTGGCGCTCACCGAGACTTGGGCCGGATCAGGAGGCTCTGGAGGCGGAGGTTCTGGAGGCTTACAGTATATCA
ATGTGGATTGTGCAAAATTAAAACGACTCCTGAAGAAAACAGCATTTAAATTTAAAGCCCTAAAGAAGGTTGCG
CAGTTAGCAGTTATAAATAGCCTGGGAGGTAGCGGAGGCGGGGGTTCCGGCGGATCACTTCTAGCTGGTCTCCC
TCTGCAGCCTGAAGAAGAAGATGGTGTGGAATTGATGGAAGCCAAATCACAGTTATTTCTTAAATACTTCACAT
TATTTATGAACCTTTTGAATGGCGGGTCTGGAGGCGGGGGCTCAGGAGGCCACCAGGAGTGTGAAGCCATTGTC
CAGTCTATCATTCATATGCGGACCCGCTGGGAACTGTCACAGCCCGACTCTATCCCCCAACACACCAAGATTGG
TGGGAGTGGGGGTGGGGGAAGCGGCGGCATATTGATCTGCAGGAATAAATTTCTTCTTAAAAATAAGCAGGCAG
ATAGAAGTTCCTTTCACTTTCTCCTTTTTTACGGGGTAGGATGTGATATTCCTTCTAGTGGAGGGGGGTCCGGA
GGCGGCGGCTCCGGGGGCGAAGGATACCTTGCAGCCACCTATCCAACTGTCGGCCAGATCAGTCCCCGAGCCAG
GAAATCCATGAGCCTGGACATGGGGCAACCTTCTCAGGCCAACACTAAGAAGTTGGGTGGATCTCTCGGAGGAG
GAGGGAGCGGCATCGTCGGCATCGTCGCAGGCCTTGCGGTTCTGGCGGTCGTTGTCATCGGAGCAGTTGTGGCT
ACTGTCATGTGCAGGCGCAAAAGCAGCGGCGGCAAAGGAGGTAGTTATTCACAGGCCGCCTCATCCGACAGTGC
CCAAGGCTCCGATGTCTCACTCACCGCG**TGA**ATTCACCATTGAGTTTAAA

Color Key:

5′ linker: TGGCTAGTTAAGCTTGGATCC
3′ linker: ATTCACCATTGAGTTTAAA

GCCACC: Kozak sequence (GCCACC**ATG**G)

MRVTAPRTLILLLSGALALTETWAGS: MHC class I signal peptide (secretion signal)

GGSGGGGSGG: GS linker

GGSLGGGGSG: GS linker

IVGIVAGLAVLAVVVIGAVVATVMCRRKSSGGKGGSYSQAASSDSAQGSDVSLTA*: MHC class I trafficking signal (MITD)
